# Midwives’ Knowledge, Attitudes, and Professional Practices Regarding Prenatal Physical Activity

**DOI:** 10.3390/healthcare13050576

**Published:** 2025-03-06

**Authors:** Émilie Brunet-Pagé, Marie-Claude Rivard, Stephanie-May Ruchat

**Affiliations:** 1 Department of Midwifery, Université du Québec à Trois-Rivières, 3351 Boulevard Des Forges, Trois-Rivières, QC G9A 5H7, Canada; 2 Groupe Interdisciplinaire de Recherche Appliquée en Santé (GIRAS), Université du Québec à Trois-Rivières, 3351 Boulevard Des Forges, Trois-Rivières, QC G9A 5H7, Canada; marie-claude.rivard@uqtr.ca (M.-C.R.); stephanie-may.ruchat@uqtr.ca (S.-M.R.); 3 Department of Human Kinetics, Université du Québec à Trois-Rivières, 3351 Boulevard Des Forges, Trois-Rivières, QC G9A 5H7, Canada

**Keywords:** attitudes, knowledge, midwifery, physical activity, pregnancy, professional practices

## Abstract

**Background/Objectives:** Prenatal physical activity (PA) offers numerous health benefits for both the mother and her child, yet few pregnant women are sufficiently active enough to obtain these benefits. Midwives play an important role in promoting prenatal PA. However, little is known about the content of the information they share with their clients regarding prenatal PA, how they communicate it, and the personal factors that might influence their counseling. In the context of prenatal PA guidance, the aim of this study was to describe the knowledge, attitudes, professional practices, and communication methods used by midwives. **Methods:** A cross-sectional descriptive study was conducted between February and June 2024 among midwives working in the Province of Quebec. An electronic questionnaire including both closed (quantitative data) and open-ended (qualitative data) questions was developed. **Results:** Fifty midwives were included in the analysis. Only 28 (56%) reported being aware of the latest Canadian guidelines for PA throughout pregnancy. The recommendations provided varied in terms of content and accuracy but were often conservative (i.e., not focused on increasing PA). Forty-five (90%) mentioned providing information on PA to their pregnant client, and eighty-four (84%) said they used bidirectional communication to share this information. The vast majority (84%) did not consider their counseling to be optimal, primarily due to a lack of training and knowledge. **Conclusions:** Our finding allowed us to gain a better understanding of current midwifery knowledge, attitudes, and professional practices regarding prenatal PA and to initiate a reflection on how to improve their knowledge, skills, and confidence in guiding their client toward prenatal PA.

## 1. Introduction

Prenatal physical activity (PA) has numerous health benefits, not only for the mother but also for the child to be. Among them, a reduction in the risk of gestational hypertensive disorders, gestational diabetes, excessive weight gain, and prenatal depression [1,2,3]. Prenatal PA also reduces this risk of macrosomia [4]. Importantly, it is not associated with an increased risk of miscarriage, congenital anomalies, or premature birth [4,5,6]. The 2019 Canadian guideline for PA throughout pregnancy recommends to every woman without medical contraindication to accumulate at least 150 min per week of moderate-intensity PA of various types [7]. However, despite the well-known health benefits of prenatal PA and existing national guideline, only 27.5% of Canadian pregnant women are active enough [8].

Midwives are autonomous professionals who provide obstetric care [9]. They recognize the importance of providing pregnant women with health information and advice [9], consider playing an important role in promoting prenatal PA, and are considered a credible source of information by women [10]. However, as highlighted by our recent scoping review [11] and that of Okafor and Goon (2021) [12], midwives are lacking knowledge and resources about prenatal PA, which adversely affect the support they provide to their clients.

In our scoping review, we were interested in the mode of communication used by midwives to support their pregnant clients regarding PA, i.e., unidirectional (information is transmitted from the midwife to the woman only) or bidirectional (information circulates between the midwife and the woman). The mode of communication is an important aspect of midwives’ support because, when it is bidirectional, it can help improve lifestyle habits, pregnancy outcomes, and client satisfaction [13,14]. Our results, based on 19 studies, showed that midwives mainly use a unidirectional mode of communication to give general recommendations on prenatal PA [11]. However, since the mode of communication was not the focus of these studies, it was identified subjectively, based on the verbs used by the authors to describe the way in which information was transmitted, such as “giving information” (unidirectional mode) or “sharing information” (bidirectional mode). Given the importance of communication when it comes to health behavior support [14], a more objective assessment of the mode of communication used by midwives is needed to draw more robust conclusions. The results of our scoping review also raised several unanswered questions about the knowledge, attitudes, and professional practices of midwives regarding prenatal PA [11]. Knowledge is defined as “a human cognitive effort to reaching the truth” [15], attitudes refer to “a state of mind that encourages a favorable or unfavorable way of being or acting” [16], while professional practices refer to “intentional, directed and potentially effective conscious actions” ([17,18] pp. 89–90). Thus, it was also important to gather information about these elements to shed light onto these questions. In the context of prenatal PA guidance, the aim of this study was to describe the knowledge, attitudes, professional practices, and communication methods used by midwives.

## 2. Materials and Methods

Population and recruitment

A convenience sample was chosen. A total of 300 midwives are registered with the Ordre des sages-femmes du Québec and it was determined that a minimum sample size of 75 (25%) would be needed to have a representative sample of the targeted population. The inclusion criteria were:

- Be a member of the Ordre des sages-femmes du Québec;
- Be under full-time or part-time contract with a healthcare facility (i.e., Centre intégré universitaire de santé et de services sociaux (CIUSSS) or Centre intégré de santé et de services sociaux (CISSS).

Recruitment was performed across the Province of Québec (Canada) through social networks and newsletters sent out by the midwifery department of the Université du Québec à Trois-Rivières, the Regroupement des sages-femmes du Québec, and the Ordre des sages-femmes du Québec, as well as through emails sent out by the main author to professional contacts.

Design and tool

A cross-sectional descriptive study was conducted between February and June 2024. An electronic questionnaire was developed in French, based on the results of our scoping review and the scientific literature. The questionnaire was tested by a small sample of people representative of the target population (n = 5) to ensure that the questions were clear and understood and to estimate the completion time. Some questions were reworded in response to feedback in order to improve their clarity for the final version.

The questionnaire included 41 questions divided into two main sections. Section 1 included questions aimed at characterizing the participants, such as age, level of education, initial training institution, number of years in practice, initial and/or continuing training on prenatal PA. Section 2 included questions on knowledge (n = 5), attitudes (n = 7), and professional practice (n = 15) regarding prenatal PA. Two types of Likert scales were used: “not very important (1)” to “very important (5)” or “strongly disagree (1)” and “strongly agree (5)”. For some items, closed-ended and open-ended questions were combined to gather more detailed information. The questionnaire was hosted on the Qualtrics survey platform (Qualtrics XM).

Statistical analyses

Descriptive statistics (mean, standard deviation and percentage) for each response were computed (IBM SPSS Statistics Version 29.0). Qualitative data from the open-ended questions were extracted and compiled in a Word document. A content analysis of the qualitative data were carried out [19]. Key elements of the qualitative data were combined with the quantitative data to enrich the understanding and present the obtained results in a more comprehensive and detailed way.

Ethical consideration

Participation was voluntary, and informed consent was obtained from all participants. This project was approved by the Research Ethics Committee of the Université du Québec à Trois-Rivières (CER-23-305-07.08).

## 3. Results

A total of 57 midwives were recruited. Six did not complete the questionnaire and one was excluded as she was not a midwife. Fifty midwives were included in the statistical analyses, including 47 who answered all the questions and three who partially completed the questionnaire. The quantitative results, supported by the qualitative results, are presented below.

Characteristics of the participants

Slightly more than half (54%) of the participants were aged between 35 and 45. Most (87%) received their initial training in midwifery at the Université du Québec à Trois-Rivières (UQTR), while the others were trained internationally in five French-speaking countries. All had a bachelor’s degree as their highest level of education. More than half (54%) had less than 10 years of practice and worked in a large urban area. Of the 14 participants who said they had received initial training in prenatal PA, many (n = 36) said it was more than five years ago. Only seven said they had received ongoing training about prenatal PA, including three who had also received hours of initial training. Further details on the participants’ characteristics are presented in Table 1.

Knowledge regarding prenatal physical activity recommendations

More than half (56%) of the participants said they were aware of recent recommendations on prenatal PA. Most (80%) thought pregnant women with no contraindications should be physically active during the first trimester of pregnancy, while several (n = 9) said it depends on the client’s symptoms (e.g., nausea, vomiting, fatigue, bleeding, risk of miscarriage). For example, one midwife said “*I recommend it at all times, unless there are contraindications such as risk of miscarriage, presence of bleeding, etc.*” (midwife number 37, MW#37). All midwives agreed that healthy women should be active during the 2nd and 3rd trimesters of pregnancy.

The participants’ recommendations on the frequency, intensity, volume, and type of PA vary widely. In terms of the recommended frequency, responses ranged from “every day” to “at least twice a week”. A moderate intensity is recommended by several participants (n = 15), and the “talk test” or a target heart rate of 120 beats/min are examples of ways provided to help pregnant women recognize a moderate-intensity PA. Few participants (n = 4) recommend an intensity “according to the level” or “the rhythm” of the client. As for the volume of PA, many (n = 33) did not answer this question, and several (n = 11) answered that they did not know what “volume” referred to. Three said that the volume of weekly PA was determined by the client’s limitations, comfort, or preferences and abilities. The participants’ recommendations regarding the type of PA are also varied. The most frequently recommended activities are yoga (n = 48), aerobic activities on land (n = 47) and in water (n = 46), and pelvic floor muscle training (n = 41). Several (n = 10) mentioned recommending muscle-strengthening exercises. Half of the participants recommended that their clients avoid some physical activities, such as weightlifting (n = 15), supine exercises (n = 14), and running (n = 12). Among prohibited PA, there are activities involving jumps or the risk of falling, such as boxing, horse riding, and skiing. To illustrate one midwife’s recommendation: “*Walk every day, 30–45 min, in addition to formal physical activities (1 to 3 times a week depending on the client’s ability and interest)*” (MW#20).

More details about the participants’ knowledge regarding prenatal PA recommendations can be found in Table S1, posted online.

Attitudes toward prenatal physical activity

Most of the participants (80%) rated prenatal PA as “very important”, while one out of five (20%) rated it as “moderately important” (mean score out of 5: 4.80 ± 0.404). In addition, more than half (52%) felt they played a “very important” role in disseminating information on prenatal PA to their clients, while 40% felt it was “moderately important” (mean score out of 5: 4.30 ± 0.891).

Many of the participants (66%) felt “comfortable” adjusting their recommendations according to the clients’ questions, context, and pre-pregnancy PA habits. One said *“[My recommendation] depends totally on their baseline level. Whether they do sport every day or not at all. I adapt according to the person I’m accompanying*” (MW#05). Another mentioned: “*It’s intrinsic to my work to adjust my recommendations so that they are individualized. If the information is not adjusted, it is unlikely to achieve its goal”* (MW#02). Several participants (n = 7) replied they did not feel comfortable adjusting their recommendations because they were not trained or did not have enough resources. For example, these two midwives commented the following: “*I’m more or less comfortable. I don’t know enough about it. (MW#16) and ‘I don’t have many resources to support my advice*” (MW#25).

Some questions were asked more specifically in relation to supporting obese pregnant women. Overall, a woman’s obesity status does not have a negative influence on the participants’ support; it remains adapted to them and not to their weight status. Two midwives explained their point of view: “*I consider that my discussion on physical activity is adapted to the person and not to her weight*” (MW#01); “*A sedentary lifestyle is much more of a problem than obesity. I provide information on physical activity regardless of the person’s weight*” (MW#08). That said, nearly half of the participants (46%) “disagreed” or “strongly disagreed” with the following statement: *I feel professionally prepared to support pregnant clients suffering from obesity in terms of prenatal physical activity* (mean score out of 5: 2.76 ± 0.902).

Of the 44 participants who answered the question *Do you consider that you provide optimal prenatal PA support to your clients?,* several (n = 7) answered “yes”, while the majority (84%) did not considered that they provide optimal support because of a lack of information, knowledge or training, or a feeling of being an imposter if their own PA level does not reach the recommendations. For example, these two midwives mentioned the following: “*I’m not necessarily up to date with the recommendations and I don’t know many tools to guide me*” (MW#28); “*In my personal life, I can’t even implement the recommendations I discuss with women. I felt like an impostor*” (MW#44). All participants thought that more initial and ongoing training, webinars, or advice from a recognized organization would enable them to offer better prenatal PA support to their clients.

More details about the participants’ attitude toward prenatal PA can be found in Table S2, posted online.

**Professional practices surrounding prenatal physical activity support**

Sharing information about prenatal physical activity

Nearly all the participants (90%) said they provide information about prenatal PA, while few (n = 5) said they do not. One of the latter qualified her statement: “*I don’t usually talk about it [prenatal PA] automatically; I answer the clients’ questions more in relation to the activities they already do*” (MW#40). The ways information is provided are varied: verbally (100%), through written documentation (22%), by referring to a PA expert (16%) or to a website (16%). Of the 45 participants who responded to the question about how often they are providing information about prenatal PA, the majority (80%) replied “frequently”, 11% “sometimes”, and 9% “at the client’s request”. In addition, of the 45 who responded the question about who starts talking about it first, many (78%) said that they address the subject first, while several (n = 10) said that it is the client who does so. Finally, when asked about how often clients asked for information about prenatal PA, several (20%) said they are asking “often”, many (68%) “occasionally”, and 12% “rarely”.

Mode of communication used to provide information about prenatal physical activity

Most of the participants (86%) mentioned using only the bidirectional mode of communication when providing information about prenatal PA, and few mentioned using either the unidirectional mode, only (8%), or both modes (6%) of communication. Of the 42 participants who used only the bidirectional mode of communication, 75% felt “fairly or moderately comfortable” doing so. Of the four participants who said they only used the unidirectional mode of communication, three felt “fairly comfortable”. Finally, the three who used both modes of communication felt “very or fairly comfortable” doing so.

Pre-screening for physical activity in pregnancy

In response to the question about the reference tool that accompanies the 2019 Canadian Guideline for physical activity throughout pregnancy, that is the *Get Active Questionnaire for Pregnancy* [20], nearly all participants (98%) said they do not use it. However, most (n = 40) said they assess previous levels of PA of their clients before providing PA recommendations. One of them explained her reasoning as follows: “*I do this assessment to adjust my advice and recommendations. So that my clients who choose to start physical activity during pregnancy find it enjoyable and continue”* (MW#17). Several (n = 6) do not assess previous levels of PA because they are not equipped, trained, or are not used to doing so. Many participants (70%) also screen for medical contraindication to PA before providing recommendations. In general, they are doing so without using any reference tools but using their personal knowledge and “common sense”, or the obstetrical record. One of these midwives said “*I don’t have a specific tool. I use my knowledge (e.g., I take into account a weak perineum, a placenta previa, a threat of premature delivery)*” (MW#19). Several participants (n = 15) do not assess contraindications to PA before providing recommendations because they do not have a tool, are not trained or lack information. Some consider that midwives do not have clients having contraindications to prenatal PA or feel that the recommendations they are providing are rather general and do not require pre-screening for PA contraindications.

Referral to a kinesiologist

Less than half of the participants (40%) answered the question about referring their clients to a kinesiologist for PA prescriptions. Of these, only five mentioned referring to a kinesiologist and doing so “rarely”. Fifteen do not refer for various reasons: they do not think about it, they do not know a kinesiologist to refer to, they consider the service too expensive, or they think their clients already consult a kinesiologist on their own. For example, this midwife said “*I don’t know any of them specifically. I don’t have any references to give them. Also, because it’s rare for women to want to invest so much time and money in physical activity during pregnancy*” (MW#18).

Barriers and facilitators to prenatal physical activity support

The lack of knowledge, skills and tools, the limited training available and being embarrassed to address the topic (e.g., because of being inactive or obese yourself) are barriers to prenatal PA support identified by the participants. Other barriers were mentioned, such as the client’s socio-economic background and her motivation towards prenatal PA. For example, one midwife said “*When they don’t seem interested or to believe in the benefits or to attach importance to prenatal PA, these are barriers to my support*” (MW#09). On the other hand, the participants identified the following factors as facilitating their PA support: having access to evidence-based data, tools (brochures, website, clinical guidelines, etc.), or training, and having the information to be shared under control.

More details about the participants’ professional practices surrounding prenatal PA support can be found in Table S3, posted online.

## 4. Discussion

This study provides a detailed description of the knowledge, attitudes and professional practices of midwives regarding prenatal PA, with an innovative approach investigating the mode of communication used when it comes to provide prenatal PA information and recommendations. Our main results show that most midwives believe that prenatal PA is very important but less think that their role in promoting it is so. They lack knowledge about the latest Canadian guidelines for PA throughout pregnancy, but nonetheless share some information, mostly using a bidirectional mode of communication, and adjust their recommendations to the client’s context. Most midwives consider prenatal PA support they are offering to their clients as sub-optimal and named several barriers, but also facilitators, to their support. Our findings, therefore, enabled us to suggest courses of action to improve the support provided by midwives to pregnant women regarding prenatal PA.

Almost all midwives consider the practice of prenatal PA to be very important; however, only half consider their role in disseminating information to be so. It is possible that midwives perceive this role as not being theirs, but rather that of PA experts, such as kinesiologists. Although kinesiologists are health professionals who specialize in PA and use movements for prevention, treatment, and performance [21], midwives play an important role in promoting healthy lifestyle habits, including prenatal PA [9]. Our findings, therefore, suggest that midwives need to be made more aware of this important role. As found in the current study, but also in our scoping review [11] and that of Okafor and Goon [12], some factors hinder this role, such as lack of time, resources, and training.

The lack of training is reflected in our results about knowledge. These show that a significant proportion of midwives have knowledge gaps regarding prenatal PA, confirming the findings of our scoping review [11] and that of Okafor and Goon (2021) [12]. As a result, they provide unclear, conflicting, and sometimes inexact information about the frequency, intensity, type and volume of PA that should be recommended to pregnant women. For example, according to international and national recommendations, moderate-intensity PA should be recommended [7,22] rather than a maximum heart rate of 120 beat per minute, as reported by one participant, which is an old recommendation from the American College of Obstetricians and Gynecologists [23]. As for the type of PA, running and supine exercise should not be systematically prohibited. The recommendation about running should be individualized. Indeed, more women are running before becoming pregnant and may choose to continue their running habits throughout pregnancy. Although most women will experience musculoskeletal pain while running during pregnancy [24], it is important to provide them support rather than telling them to avoid running. As for supine exercise, midwives should instead recommend to pregnant women who are experiencing lightheadedness, nausea, or who are feeling unwell when they exercise flat on their back to modify their exercise position [25]. These examples illustrate inexact information shared by participants that are based on outdated prenatal PA recommendations [23,26] and that may induce fear of certain activities or types of exercise. Importantly, providing conflicting information from healthcare professionals has been identified as a barrier to prenatal PA [27]. Therefore, it is important that midwives provide the most up-to-date evidence-based information to their client and avoid unclear and conflicting information to successfully support them toward regular practice of PA [28]. Nevertheless, midwives recommend a variety of cardiovascular PA, as well as pelvic floor muscle training, in line with national recommendations [7]. However, muscle strengthening exercises are poorly recommended. Midwives may consider again that it is part of the kinesiologists’ expertise to recommend this more specific type of exercise.

Based on some of our findings, we would have expected midwives to refer their clients to a kinesiologist to offer them more specific support toward prenatal PA. However, our results show that very few of them are inclined to do so because they do not know a kinesiologist or because they think their clients already consult them or do not want to invest time and money in this specialist. This finding, also raised by Walker’s et al. (2019) in the context of prenatal and postnatal weight management, highlights a wider and pervasive issue within the midwifery community, where there is insufficient interprofessional collaboration to promote healthy behaviors such as prenatal PA [29]. As emphasized by Hyvärinen and colleagues (2022) [30], promoting prenatal PA should be based on an interdisciplinary approach, involving several health professionals. Overall, these findings show that there is a need to raise awareness of the importance and benefits of multidisciplinary care; midwives should know when and to which professionals to refer their clients who need support from a specialist, whether in PA or another health area (e.g., nutrition, mental health, pelvic health, and musculoskeletal health), to ensure optimal care and service provision.

It is recognized that midwives provide pregnancy-centered care [9], which implies that they « focus on the woman’s individual needs, aspirations and expectations, rather than on the needs of the institution or professionals » [31] (p. 12). Our results show that in some respects, midwives focus on specific needs of their client, as most of them mentioned assessing previous levels of PA of their clients before providing PA recommendations, feeling comfortable adjusting their recommendations according to the client’s context and using a bidirectional mode of communication, considered optimal when it comes to discussing behavior change. These are major professional strengths, showing a willingness to engage in genuine discussion with the clients and provide patient-centered care and undeniable assets for facilitating changes in behavior regarding prenatal PA [28]. Nevertheless, most midwives consider offering a sub-optimal prenatal PA support to their client and certain barriers have been raised. For example, the feeling of being an imposter when it comes to provide information on prenatal PA was a concern and seems to hinder possible discussions. Indeed, discomfort arises when the midwife’s personal PA level does not meet national recommendations or when she is overweight or obese. This feeling has also been reported by nurses who consider that they should be role models for health behaviors, including PA, and who find it difficult to promote healthy lifestyle habits, or see their credibility affected, when they themselves do not meet PA recommendations or are overweight or obese [32,33]. In addition, some midwives consider it more difficult to motivate clients whose socio-economic background is unfavorable, who were not active before becoming pregnant, or who are overweight or obese. Offering ongoing training in motivational interviewing could be an interesting avenue that would enable midwives to better support changes in PA behavior in pregnant women who may need more sustained support [34].

In the light of our results, midwives need better initial training and access to ongoing education to gain knowledge and keep their knowledge up-to-date and improve their skills and confidence to provide prenatal PA support. In this respect, Hyvärinen and colleagues (2022) [30] showed that an educational program offered to midwifery students and aimed at improving PA counseling was highly successful. Midwives also need better knowledge of existing resources in prenatal PA and where to find them. In fact, these are key elements identified by our participants as facilitating their support towards prenatal PA.

It is recognized that the work environment influences midwives’ support and advice regarding health promotion through PA [33]. Creating a work environment that helps keep knowledge up-to-date and favors evidence-based clinical practice would enable midwives to fully fulfill their role of promoting and supporting prenatal PA. One of the strategies would be to designate a “champion” midwife in the team whose leadership is recognized by her peers who will ensure dissemination of up-to-date clinical practice guidelines, access to a list of other local healthcare professionals, such as kinesiologists, to resources, such as brochures, pamphlets to distribute and websites to recommend, as well as to standardized forms to assess, promote, and follow prenatal PA practice of clients. The university, or institution, providing initial training, the professional order, the teams of midwives, as well as midwives themselves should also commit to favoring this type of environment, to ensure that the scientific evidence and clinical resources are shared and updated on an ongoing basis. Pregnant women would fully benefit from optimal support focused on their prenatal PA needs and thus would enjoy all the benefits of PA for their own health and that of their future child. Better knowledge of the women’s perceptions of the prenatal PA support they receive from their midwife would make it possible to draw up a more comprehensive picture of clinical practice so that we can prioritize actions to support midwives while meeting pregnant women’s expectations.

### Strength and Limitations

The current study is one of the few to examine clinical practice of midwives in a Quebec context and document the mode of communication used to provide information on prenatal PA. Our study has several strengths that deserve to be mentioned. First, it is based on knowledge gaps identified in our recent scoping review [11], which guarantees its quality and rigor as well as its social relevance. Second, the questionnaire used to expand the scientific literature on the knowledge, attitudes, and professional practices of midwives was custom-developed and validated by a sub-sample of midwives. However, certain limitations must also be raised. Although several strategies were used to disseminate our study and reach as many midwives as possible, (a potential total of >300), only 50 midwives were recruited. This represents around 16% of the target population, which is less than the desired 25%. In addition, a possible selection bias exists as it is likely that midwives with a strong interest in prenatal PA were more likely to participate in the study. These limitations can therefore affect the external validity of our study. Moreover, a social desirability bias cannot be ruled out since the principal investigator is well known in the midwifery community in Quebec. However, the questionnaire was totally anonymous and proper instructions were given to ensure the most honest answers were provided. Lastly, because of the small sample, it was not possible to carry out more detailed analyses to identify elements that might characterize more favorable knowledge, attitudes, and professional practices.

## 5. Conclusions

Our finding allowed us to gain a better understanding of current midwifery knowledge, attitudes, and professional practices regarding prenatal PA and to initiate a reflection on how to improve their knowledge, skills, and confidence in supporting their client toward prenatal PA. Improving initial training and access to ongoing education and providing motivation by management staff may be an avenue, with the hope that this will lead to regular PA practice in clients and thus healthy pregnancies and well-prepared childbirth.

## Figures and Tables

**Table 1:** Characteristics of the 50 participants included in the study.

Variables	Mean ± SD (min–max); or n; %
**Age (years old)**	39.8 ± 8.3 (24–60)
<25	2
25 to 34	11
35 to 44	26
≥45	11
**Country (institution) of initial training in midwifery**	
Canada (UQTR)	43; 86
Others:	7; 14
France	2; 4
Iran	2; 4
Lebanon	1; 2
Belgium	1; 2
Switzerland	1; 2
**Highest level of education in midwifery**	
Bachelor’s degree	44; 88
Master’s degree	6; 12
**Number of years in midwifery clinical practice**	
<5	11; 22
5 to 10	16; 32
11 to 20	20; 40
>20	2; 4
Missing response	1; 2
**Description of primarily practice location**	
Remote	6; 12
Rural (N < 1000 people)	0; 0
Small population centers (N = 1000 to 29,999)	1; 2
Medium population centers (N = 30,000 to 99,999)	15; 30
Large urban population centers (N ≥ 100,000).	27; 54
Other: First Nation community, remote region in North of Quebec	1; 2
**Initial training in prenatal physical activity**	
No	17; 34
Yes	14; 28
I don’t remember	19; 38
**Number of training hours**	
<1 h	3; 22
1 h to 3 h	2; 14
>3 h to 6 h	2; 14
I don’t remember	7; 50
**When training was done (years ago)**	
1 to 3	1; 7
4 to 5	3; 21
>5	10; 72
**Ongoing training in prenatal physical activity**	
No	43; 86
Yes	7; 14
**Number of training hours**	
<1 h	2; 29
1 h to 3 h	2; 28
>3 h to 6 h	3; 43
**When training was done (years ago)**	
1 to 3	4; 57
4 to 5	0; 0
>5	3; 423
**Consider yourself as physically active**	
Yes	35; 70

UQTR: Université du Québec à Trois-Rivières.

## Data Availability

The original contributions presented in this study are included in the article/Supplementary Material. Further inquiries can be directed to the corresponding author.

## Supplementary Materials

The following supporting information can be downloaded at: https://www.mdpi.com/article/10.3390/healthcare13050576/s1, Table S1: Key results about midwives’ knowledge regarding prenatal physical activity recommendations; Table S2: Key results about midwives’ attitudes toward prenatal physical activity; Table S3: Key results about midwives’ professional practices surrounding prenatal physical activity support.
