# Supplementary material for: Midwives’ Knowledge, Attitudes, and Professional Practices Regarding Prenatal Physical Activity
Source: Healthcare (Basel). 2025 Mar 6;13(5):576. doi: 10.3390/healthcare13050576 (PMC11899359; doi:10.3390/healthcare13050576)
Supplement: Supplementary file 1 [file healthcare-13-00576-s001.zip › healthcare-3352825-supplementary.pdf]

Table S1: Key results about midwives' knowledge regarding prenatal physical activity recommendations.

**Do you think you are aware of the most recent recommendations (from the World Health Organisation or the Society of Obstetricians and Gynaecologists of Canada) concerning prenatal physical activity?**	**Yes** : 28 (56%)<br>**No** : 22 (44%)
**What frequency, duration, intensity and/or volume of physical activity would you typically recommend for pregnant clients?**	**Frequency (n=number of times reported):**<br>3 times a week (8)<br>Every day (9)<br>3 to 4 times a week (5)<br>3 to 5 times a week (3)<br>120 minutes per week<br>2 hours per week<br>At least 2 times a week<br><br>**Duration of activity:**<br>"Depending on intensity"<br>"At least 20 minutes, able to talk but not sing. Check heart rate if needed, aiming for 120 bpm, 150 minutes per week."<br><br>**Intensity (n=number of times reported):**<br>Low to moderate (5)<br>Moderate or average (16)<br>Average to high (1)<br>According to one's level or at one's own pace (4)<br>Able to talk but not sing (3)<br>Check heart rate if needed, aiming for 120 bpm (1)<br>Normal (1)<br><br>**Volume:**<br>"I don't understand" or "I don't know" (11)<br>According to one's limits, preferences, comfort (3)<br>150 minutes (2)<br>Varies (1)<br><br>**Additional Information**<br><br>« It totally depends on their baseline level. Whether they exercise daily or not at all. I adapt based on the individual I'm supporting. »
**What type of exercise would you recommend to your pregnant patients (please check all that apply)?**	Response choices:<br><br>Yoga: 48 (96%)<br>Aerobic on land (e.g., walking): 47 (94%)<br>Aerobic in water (e.g., swimming): 46 (92%)<br>Pelvic floor muscle training: 41 (82%)<br>Resistance training (e.g., lifting weights): 10 (20%)<br>Other: walking, spinning, TRX, training on a ball, dancing, stretching, cycling, Pilates

	I generally do not recommend specific types of activities to my pregnant clients: 0
**Are there exercises that you recommend that pregnant women avoid during pregnancy?**	**Yes**: 25 (50%)<br>**No**: 25 (50%)<br><br>**If yes, what type of prenatal physical activity would you recommend your clients avoid?**<br>Response choices:<br><br>Heavy weightlifting: 15 (60%)<br>Supine exercise: 14 (56%)<br>Valsalva maneuveur : 0<br>Balistic movements : 0<br>Running: 12 (48%)<br>Other:<br>"I recommend avoiding all exercises that engage the rectus abdominis (e.g., sit-ups) starting from the second trimester."<br>"No abdominal exercises during pregnancy, it is not recommended."<br>"Any activity involving jumps (do not start during pregnancy, okay to continue if already accustomed, while listening to your body) or a risk of falling (ice skating, alpine skiing), unless already accustomed, and with precautions due to the change in center of gravity and increased joint hyperlaxity/mobility."

Table S2: Key results about midwives' attitudes toward prenatal physical activity.

**On a scale from 1-5 where 1 is not important and 5 is very important, how would you rate the importance of physical activity during pregnancy?**	1 - not important: 40 (80%)<br>2 - slightly important:10 (20%)<br>3 - neutral: 0<br>4 - moderately important: 0<br>5 - very important: 0
**On a scale from 1-5 where 1 is not important and 5 is very important, how important do you believe your role is in providing prenatal physical activity information to your clients?**	1 - not important: 26 (52 %)<br>2 - slightly important: 20 (40%)<br>3 - neutral: 2 (4%)<br>4 - moderately important: 2 (4%)<br>5 - very important: 1 (2%)
**On a scale from 1-5 where 1 is not comfortable at all and 5 is very comfortable, how comfortable are you providing prenatal physical activity information when you use a unidirectional mode of communication with your clients?**	1 - not comfortable at all: 3 (6%)<br>2 - a little comfortable: 19 (40%)<br>3 - moderately comfortable: 13 (27 %)<br>4 - mostly comfortable: 9 (19%)<br>5 - very comfortable: 4 (8%)<br>*Note*: 2 MW did not respond to the question
**On a scale from 1-5 where 1 is not comfortable at all and 5 is very comfortable, how comfortable are you providing prenatal physical activity information when you use a bidirectional mode of communication with your clients?**	1 - not comfortable at all: 0<br>2 - a little comfortable: 3 (6%)<br>3 - moderately comfortable: 6 (12 %)<br>4 - mostly comfortable: 29 (58 %)<br>5 - very comfortable: 10 (20%)<br>*Note*: 2 MW did not respond to the question
**Do you feel comfortable adjusting your prenatal physical activity recommendations according to the context, social determinants, and risk factors of your clients?**	**Yes**: 33 (82,5 %)<br>**No**: 7 (17,5 %)<br>*Note*: 10 MW did not respond to the question<br><br>**Yes, why?**<br>"It's intrinsic to my job to adjust my recommendations so that they are individualized. If the information is not tailored, it is unlikely to achieve its goal."<br>Personal experience in physical activity<br><br>**No, why?**<br>Lack of knowledge<br>I'm not a kinesiologist<br>Many barriers for women in prenatal physical activity<br>I don't feel sufficiently trained<br>Not enough resources (tools)
**I feel professionally prepared to support pregnant clients with obesity towards prenatal physical activity.**	1 (strongly disagree): 0<br>2 (disagree): 13 (26%)<br>3 (neutral): 13 (26%)<br>4 (agree): 21 (42%)<br>5 (strongly agree): 2 (4%)<br>*Note*: 1 MW did not respond to the question

Table S3: Key results about midwives' professional practices surrounding prenatal physical activity support.

**Do you provide information about prenatal physical activity to your clients in your clinical practice?**	**Yes**: 45 (90%)<br><br>Rarely: 0<br>On occasion: 0<br>Sometimes: 5 (11 %)<br>Frequently: 36 (80 %)<br>At the patient request: 4 (9 %)<br>In case of a medical condition: 0<br><br>**No**: 5 (10 %)<br><br>**Additional Information from those who provides information.**<br><br>"When I'm asked the question or for specific cases (requiring more movement). Otherwise, I'm not necessarily inclined to talk about it."<br>"To continue the activity she's practicing safely (avoid falls) and to respect her pace, which changes with pregnancy."<br>"I'm not used to talking about it automatically in general; I tend to respond more to the questions clients ask related to the activities they are already doing."<br>"It depends. If they ask me about a specific sport, I ask what they're used to doing before giving my recommendations, but I don't do this systematically."<br>"I don't encourage starting a new high-intensity activity, but rather the continuation of what they were doing, with the necessary adaptations. Otherwise, I refer them to various online resources or local resources (such as Bedon Bout'chou, which has a diverse offering)."<br><br>**Additional Information from those who don't provide information.**<br><br>"I don't feel equipped to do so."<br>"I don't think I'm trained to assess their level.<br>"To continue the activity she's practicing safely (avoid falls) and to respect her pace, which changes with pregnancy."<br>"I mainly go with general advice, unless the woman brings up the topic herself and wants to know if she can continue a particular activity."<br>"When I'm asked the question or for specific cases (requiring more movement). Otherwise, I'm not necessarily inclined to talk about it."
If you provide information on prenatal physical activity to your clients, who usually brings up this subject first?	You: 35 (78 %)<br>Your client: 10 (22 %)
How often do your clients ask you for information about prenatal physical activity?	Often : 10 (22%)<br>Occasionally : 34 (68%)<br>Rarely : 6 (12%)

**How often do you get asked for advice about physical activity in pregnancy?**	Never: 0<br>Rarely: 6 (12 %)<br>Occasionally: 34 (68%)<br>Often: 10 (20 %)
**How do you provide prenatal physical activity information to your clients?**	Verbally: 50 (100%)<br>Pamphlet/booklet: 11 (22 %)<br>Directed to website: 8 (16 %)<br>If yes, which websites are you directing your patients to?<br>*Naître et grandir*<br>*Bougeote et placotine*<br>Youtube (online prenatal yoga)<br><br>Refer to an expert in prenatal exercise: 8 (16 %)<br>Does not apply as I am no giving information about prenatal PA to my clients: 0
**When you provide verbal information about prenatal physical activity, which mode of communication do you use?**	Unidirectional mode of communication: 4 (8 %)<br>Bidirectional mode of communication): 42 (86 %)<br>Both mode of communication: 3 (6%)<br>*Note*: 1 MW did not respond to the question
**Do you assess your clients' current and or previous activity level before providing physical activity recommendations?**	**Yes**: 40 (89 %)<br>**No**: 5 (11 %)<br>*Note*: 5 MW did not respond to the question<br><br>**More information from those who responded "yes"**<br><br>**"To adjust my advice and recommendations.**<br>**So that my clients who choose to initiate physical activity during pregnancy can enjoy it and continue."**<br>**"I recommend maintaining the same level of physical activity as before pregnancy (if the client is sedentary, it's not the time to train for a marathon, for example), but I also ask about which sports are practiced to ensure it's done safely.**<br>**Unless the client wasn't active before, in which case we discuss ways to move more."**<br>**"Evaluate might be too strong a word for what I do: I ask them about the physical activity they practice (type, frequency) to see if they can continue, adapt... or start gently!"**
**Do you screen your clients for contraindication to prenatal physical activity before providing them recommendations on prenatal physical activity?**	**Yes**: 31 (70 %)<br>**No**: 13 (30 %)<br>*Note*: 6 MW did not respond to the question
**In your practice, do you, or did you, take care of obese clients?**	**Yes**: 49 (100%)<br>**No**: 0<br>*Note*: 1 MW did not respond to the question
**If a client presents obesity, does it influence your prenatal physical activity support?**	**Yes**: 19 (41%)<br><br>**More information from those who responded "yes"**<br><br>**"I approach the subject in the same way. A woman with obesity can be very active." However, I also discuss the effects of obesity on pregnancy (e.g., diabetes, hypertension) and how physical activity can have a protective effect.**

	**"I will encourage exercise immediately after meals to support better blood sugar levels."**<br>**"I will talk about it more..."**<br><br><br><br><br><br>**No**: 27 (59%)<br><br>**More information from those who responded "no"**<br><br>"I believe that my discussion about physical activity is tailored to the individual, not their weight. I won't recommend more to an overweight person than I would to someone with what is considered a healthy weight or someone underweight."<br>"I don't want them to feel judged."<br>"Weight is not necessarily related to physical activity."<br>"Sedentarism is much more problematic than obesity. I provide information about physical activity regardless of the person's weight. I always start by asking about the person's lifestyle habits before discussing physical activity."<br><br>*Note*: 4 MW did not respond to the question
**Do you use the Get Active Questionnaire for Pregnancy in your practice?**	**Yes**: 1 (2%)<br>**No**: 47 (98 %)<br>*Note*: 2 MW did not respond to the question
**In your professional practice, do you take into account of the social determinants of health (contexts and living conditions) when guiding your clients towards prenatal physical activity?**	**Yes**: 45 (94%)<br>**No**: 3 (6 %)<br>*Note*: 2 MW did not respond to the question
**Is a kinesiologist available to support pregnant clients in your area?**	**Yes**: 20 (42%)<br>**No**: 5 (10%)<br>**Do not know**: 23 (48%)<br>*Note*: 2 MW did not respond to the question
**Do you refer your pregnant clients to a kinesiologist to prescribe prenatal physical activity?**	Of the 20 MW who mentioned that a kinesiologist was available/accessible in their area:<br>**Yes**: 5 (25%)<br><br>Always: 0<br>Sometimes: 0<br>Rarely: 5 (100%)<br><br>**No**: 15 (75%)<br><br>**If not, why**:<br><br>**Too expensive, lack of knowledge about kinesiologists, I don't think of it, I don't know any, women consult kinesiologists on their own without a referral from me.**

	**"I've never thought of this option. I refer many women to pelvic floor physiotherapists, but not a kinesiologist for prenatal physical activity."**
**Have you already identified certain factors that make it easier for you to offer your clients advice on prenatal physical activity?**	**Having evidence-based data**<br>**Personal interest in physical activity**<br>**Knowing the client's context**<br>**When clients are interested, open, and ask questions**<br>**The client's motivation**<br>**Having brochures, websites to refer to**<br>**Appointment time (1 hour and general)**<br>**Having concise information to share**<br>**Being a preceptor and having a student who is up-to-date with the latest recommendations**<br>**Clinical guidelines**<br>**Free classes, online courses**<br>**Having a "pre-written" note, with the minimal elements to share and adapt based on the discussion**<br>**The client's socio-economic status, their environment, and positive relationships within the couple**
**Have you already identified certain barriers that hinder your support for prenatal physical activity with your clients?**	**Lack of time**<br>**Lack of client interest**<br>**Lack of knowledge**<br>**Lack of tools**<br>**No kinesiologist available in the area**<br>**Sensitive topic when the pregnant woman is overweight**<br>**My discomfort in addressing the topic of physical activity with obese women**<br>**My own overweight**<br>**My embarrassment in addressing the topic**<br>**The client's socio-economic status**<br>**My impression of not mastering the subject**<br>**A history of eating disorders or a woman who has experienced grossophobia**
**What would help you to feel more comfortable at providing information about exercise during pregnancy?**	Response choices:<br><br>More training in residency: 44 (94 %)<br>more guidance from Society of Obstetricians and Gynaecologists of Canada: 24 (51%)<br>Seminars: 15 (32%)<br>Infographics: 10 (21 %)<br>Pamphlets: 18 (38%)<br>Short videos: 25 (53%)<br>Other: networking<br>*Note*: 3 MF did not respond to the question
